# The open abdomen in trauma and non-trauma patients: WSES guidelines

**DOI:** 10.1186/s13017-018-0167-4

**Published:** 2018-02-02

**Authors:** Federico Coccolini, Derek Roberts, Luca Ansaloni, Rao Ivatury, Emiliano Gamberini, Yoram Kluger, Ernest E. Moore, Raul Coimbra, Andrew W. Kirkpatrick, Bruno M. Pereira, Giulia Montori, Marco Ceresoli, Fikri M. Abu-Zidan, Massimo Sartelli, George Velmahos, Gustavo Pereira Fraga, Ari Leppaniemi, Matti Tolonen, Joseph Galante, Tarek Razek, Ron Maier, Miklosh Bala, Boris Sakakushev, Vladimir Khokha, Manu Malbrain, Vanni Agnoletti, Andrew Peitzman, Zaza Demetrashvili, Michael Sugrue, Salomone Di Saverio, Ingo Martzi, Kjetil Soreide, Walter Biffl, Paula Ferrada, Neil Parry, Philippe Montravers, Rita Maria Melotti, Francesco Salvetti, Tino M. Valetti, Thomas Scalea, Osvaldo Chiara, Stefania Cimbanassi, Jeffry L. Kashuk, Martha Larrea, Juan Alberto Martinez Hernandez, Heng-Fu Lin, Mircea Chirica, Catherine Arvieux, Camilla Bing, Tal Horer, Belinda De Simone, Peter Masiakos, Viktor Reva, Nicola DeAngelis, Kaoru Kike, Zsolt J. Balogh, Paola Fugazzola, Matteo Tomasoni, Rifat Latifi, Noel Naidoo, Dieter Weber, Lauri Handolin, Kenji Inaba, Andreas Hecker, Yuan Kuo-Ching, Carlos A. Ordoñez, Sandro Rizoli, Carlos Augusto Gomes, Marc De Moya, Imtiaz Wani, Alain Chichom Mefire, Ken Boffard, Lena Napolitano, Fausto Catena

**Affiliations:** 1 General Emergency and Trauma Surgery, Bufalini Hospital, Viale Giovanni Ghirotti, 286, 47521 Cesena, Italy; 2 Department of Surgery, Foothills Medical Centre, Calgary, Canada; 3 Virginia Commonwealth University, Richmond, VA USA; 4 ICU Department, Bufalini Hospital, Cesena, Italy; 5 Division of General Surgery, Rambam Health Care Campus, Haifa, Israel; 6 Trauma Surgery, Denver Health, Denver, CO USA; 7 Department of Surgery, UC San Diego Health System, San Diego, USA; 8 Faculdade de Ciências Médicas (FCM)–Unicamp Campinas, Campinas, SP Brazil; 9 Department of Surgery, College of Medicine and Health Sciences, UAE University, Al-Ain, United Arab Emirates; 10 Department of Surgery, Macerata Hospital, Macerata, Italy; 11 Department of Trauma, Emergency Surgery and Surgical Critical Care, Massachusetts General Hospital, Boston, MA USA; 12 Second Department of Surgery, Meilahti Hospital, Helsinki, Finland; 13 Trauma and Acute Care Surgery and Surgical Critical Care Trauma, Department of Surgery, University of California, Davis, USA; 14 General and Emergency Surgery, McGill University Health Centre, Montréal, QC Canada; 15 Department of Surgery, Harborview Medical Centre, Seattle, USA; 16 General Surgery Department, Hadassah Medical Centre, Jerusalem, Israel; 17 First Clinic of General Surgery, University Hospital/UMBAL/St George Plovdiv, Plovdiv, Bulgaria; 18 General Surgery, Mozir Hospital, Mozir City, Belarus; 19 ICU and High Care Burn Unit, Ziekenhius Netwerk Antwerpen, Antwerpen, Belgium; 20 Department of Surgery, Trauma and Surgical Services, University of Pittsburgh School of Medicine, Pittsburgh, USA; 21 Department of Surgery, Tbilisi State Medical University, Kipshidze Central University Hospital, Tbilisi, Georgia; 22 General Surgery Department, Letterkenny Hospital, Letterkenny, Ireland; 23 Addenbrooke’s Hospital, Cambridge, UK; 24 Klinik für Unfall-, Hand- und Wiederherstellungschirurgie Universitätsklinikum Goethe-Universität Frankfurt, Frankfurt, Germany; 25 Department of Clinical Medicine, University of Bergen, Bergen, Norway; 26 Department of Gastrointestinal Surgery, Stavanger University Hospital, Stavanger, Norway; 27 Acute Care Surgery, The Queen’s Medical Center, Honolulu, HI USA; 28 General and Trauma Surgery Department, London Health Sciences Centre, Victoria Hospital, London, ON Canada; 29 Département d’Anesthésie-Réanimation, CHU Bichat Claude-Bernard-HUPNVS, Assistance Publique-Hôpitaux de Paris, University Denis Diderot, Paris, France; 30 ICU Department, Sant’Orsola-Malpighi University Hospital, Bologna, Italy; 31 ICU Department, Papa Giovanni XXIII Hospital, Bergamo, Italy; 32 Surgery Department, University of Maryland School of Medicine, Baltimore, MD USA; 33 Emergency and Trauma Surgery Department, Niguarda Hospital, Milano, Italy; 34 General Surgery Department, Assuta Medical Centers, Tel Aviv, Israel; 35 General Surgery, “General Calixto García”, Habana Medicine University, Havana, Cuba; 36 General Surgery, Medical Faculty “General Calixto Garcia”, Habana Medicine University, Havana, Cuba; 37 Division of Trauma, Department of Surgery, Far-Eastern Memorial Hospital, New Taipei City, Taiwan, Republic of China; 38 Clin. Univ. de Chirurgie Digestive et de l’Urgence, CHUGA-CHU Grenoble Alpes UGA-Université Grenoble Alpes, Grenoble, France; 39 General and Emergency Surgery Department, Empoli Hospital, Empoli, Italy; 40 Department of Cardiothoracic and Vascular Surgery, Örebro University Hospital and Örebro University, Orebro, Sweden; 41 General Surgery, Perpignan Hospital, Perpignan, France; 42 Pediatric Trauma Service, Massachusetts General Hospital, Boston, MA USA; 43 General and Emergency Surgery, Sergei Kirov Military Academy, Saint Petersburg, Russia; 44 Unit of Digestive Surgery, HPB Surgery and Liver Transplant, Henri Mondor Hospital, Créteil, France; 45 Department of Primary Care and Emergency Medicine, Kyoto University Graduate School of Medicine, Kyoto, Japan; 46 Department of Traumatology, John Hunter Hospital and University of Newcastle, Newcastle, NSW Australia; 47 General Surgery Department, Westchester Medical Center, Westchester, NY USA; 48 Department of Surgery, University of KwaZulu-Natal, Durban, South Africa; 49 Department of General Surgery, Royal Perth Hospital, The University of Western Australia & The University of Newcastle, Perth, Australia; 50 Trauma Unit, Helsinki University Hospital, Helsinki, Finland; 51 Division of Trauma and Critical Care, LAC+USC Medical Center, University of Southern California, California, Los Angeles USA; 52 General and Thoracic Surgery, Giessen Hospital, Giessen, Germany; 53 Acute Care Surgery and Traumatology, Taipei Medical University Hospital, Taipei City, Taiwan, Republic of China; 54 Trauma and Acute Care Surgery, Fundacion Valle del Lili, Cali, Colombia; 55 Trauma and Acute Care Service, St Michael’s Hospital, Toronto, ON Canada; 56 Hospital Universitário Terezinha de Jesus, Faculdade de Ciências Médicas e da Saúde de Juiz de Fora (SUPREMA), Juiz de Fora, Brazil; 57 Trauma, Acute Care Surgery, Medical College of Wisconsin/Froedtert Trauma Center, Milwaukee, WI USA; 58 Department of Surgery, Sheri-Kashmir Institute of Medical Sciences, Srinagar, India; 59 Department of Surgery and Obs/Gyn, Faculty of Health Sciences, University of Buea, Buea, Cameroon; 60 Milpark Hospital Academic Trauma Center, University of the Witwatersrand, Johannesburg, South Africa; 61 Acute Care Surgery, Department of Surgery, University of Michigan Health System, Ann Arbor, MI USA; 62 Emergency and Trauma Surgery, Parma Maggiore Hospital, Parma, Italy

**Keywords:** Open abdomen, Laparostomy, Non-trauma, Trauma, Peritonitis, Pancreatitis, Vascular emergencies, Intra-abdominal infection, Fistula, Nutrition, Re-exploration, Reintervention, Closure, Biological, Synthetic, Mesh, Technique, Timing, Guidelines

## Abstract

Damage control resuscitation may lead to postoperative intra-abdominal hypertension or abdominal compartment syndrome. These conditions may result in a vicious, self-perpetuating cycle leading to severe physiologic derangements and multiorgan failure unless interrupted by abdominal (surgical or other) decompression. Further, in some clinical situations, the abdomen cannot be closed due to the visceral edema, the inability to control the compelling source of infection or the necessity to re-explore (as a “planned second-look” laparotomy) or complete previously initiated damage control procedures or in cases of abdominal wall disruption. The open abdomen in trauma and non-trauma patients has been proposed to be effective in preventing or treating deranged physiology in patients with severe injuries or critical illness when no other perceived options exist. Its use, however, remains controversial as it is resource consuming and represents a non-anatomic situation with the potential for severe adverse effects. Its use, therefore, should only be considered in patients who would most benefit from it. Abdominal fascia-to-fascia closure should be done as soon as the patient can physiologically tolerate it. All precautions to minimize complications should be implemented.

## Background

Damage control management (DCM) of severely injured or physiologically deranged patients is considered by many to consist of damage control resuscitation (DCR) and damage control surgery (DCS). Use of DCM in patients with deranged physiology may trigger intra-abdominal hypertension (IAH) or abdominal compartment syndrome (ACS) that may aggravate physiologic derangement or multiorgan failure (MOF) in a vicious circle unless interrupted by abdominal decompression (surgical or other) [1, 2]. Further, in other clinical situations, the abdomen cannot be closed due to visceral edema, the inability to completely control the compelling source of infection or to the necessity to re-explore (in a “planned re-look laparotomy”) or to complete DCS procedures or in cases of abdominal wall damage. Although open abdomen (OA) has been proposed to be effective in preventing or treating deranged physiology in patients with severe injuries or critical illness, it must be recognized as a non-anatomic situation that has potential for severe side effects while increasing resource utilization [3].

**Fig. 1:**
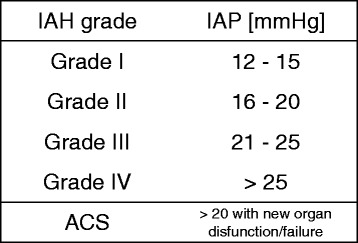
WSACS grading of intra-abdominal hypertension (IAH) (IAP intra-abdominal pressure, ACS abdominal compartment syndrome) [4]

The World Society for Emergency Surgery (WSES) accepted the definitions of IAH, ACS, and related conditions published by the World Society Abdominal Compartment Syndrome in 2013 (WSACS) [2–4] (Fig. 1).

OA management consists of intentionally leaving the abdominal fascial edges of the paired rectus abdominus muscles un-approximated (laparostomy) in order to truncate operation, prevent IAH/ACS, and facilitate re-exploration without damaging the abdominal fascia [3]. Temporary abdominal closure (TAC) refers to the method for providing protection to the abdominal viscera during the time the fascia remains open [2, 5]. Patients undergoing OA management are at risk of developing entero-atmospheric fistula (EAF) and a “frozen abdomen,” intra-abdominal abscesses, and lower rates of definitive fascial closure [6, 7]. The risk-benefit ratio must be kept in mind in using OA. It should not be performed liberally. Measures to mitigate complications are necessary. In all patients with an OA, every effort should be exerted to achieve primary fascial closure (i.e., fascia-to-fascia closure of the abdominal wall within the index hospitalization) as soon as the patient can physiologically tolerate it [3].

### Purpose and use of this guideline

The guidelines are evidence-based, with the grades of recommendation, based on the evidence. These guidelines present methods for optimal management of open abdomen in trauma and non-trauma patients. They do not represent a standard of practice. They are suggested plans of care, based on best available evidence and a consensus of experts. They, however, do not exclude other approaches as being within a standard of practice. For example, they should not be used to compel adherence to a given method of medical management, which should be finally determined after taking into account conditions at the relevant medical institution (staff levels, experience, equipment, etc.) and the characteristics of the individual patient. The responsibility for the results, however, rests with the engaging practitioners and not aged therein, and not the consensus group.

## Methods

A computerized search was performed in MEDLINE, EMBASE, and Scopus by an information scientist/librarian for the time range of January 1980 to August 2017. The terms open abdomen, laparostomy, injuries, trauma, peritonitis, pancreatitis, vascular, ischemia, resuscitation, adult, management, infection, intensive care unit, anastomosis, vasopressors, and follow-up in various combinations with the use of the Boolean operators “AND” and “OR” were used. No search restrictions were imposed. The dates were selected to allow comprehensive published abstracts of clinical trials, consensus conferences, comparative studies, congresses, guidelines, government publications, multicenter studies, systematic reviews, meta-analyses, large case series, original articles, and randomized controlled trials. Case reports and small case series were excluded. We also analyzed the reference lists of relevant narrative review articles identified during the search to identify any studies that may have been missed.

For each article, we subsequently applied a level of evidence (LE) using the Grading of Recommendations, Assessment, Development, and Evaluation (GRADE) system [8] (Table 1). The full GRADE process was not used, as this system is difficult to apply when scant evidence exists. A group of experts in the field of OA management, coordinated by a central coordinator, were subsequently convened in order to elicit their evidence-based opinions on certain key clinical questions relating to the OA. Through a Delphi process, the clinical questions were discussed in rounds. The central coordinator assembled the different answers derived from each round. Each version was then revised and improved through iterative evaluation. The final version about which the agreement was reached resulted in the comments and recommendations made in the present guideline. Statements have been summarized in Table 2.

**Table 1:** GRADE system to evaluate the level of evidence and recommendation

Grade of recommendation	Clarity of risk/benefit	Quality of supporting evidence	Implications
1A			
Strong recommendation, high-quality evidence	Benefits clearly outweigh risk and burdens, or vice versa	RCTs without important limitations or overwhelming evidence from observational studies	Strong recommendation, applies to most patients in most circumstances without reservation
1B			
Strong recommendation, moderate-quality evidence	Benefits clearly outweigh risk and burdens, or vice versa	RCTs with important limitations (inconsistent results, methodological flaws, indirect analyses, or imprecise conclusions) or exceptionally strong evidence from observational studies	Strong recommendation, applies to most patients in most circumstances without reservation
1C			
Strong recommendation, low-quality or very low-quality evidence	Benefits clearly outweigh risk and burdens, or vice versa	Observational studies or case series	Strong recommendation but subject to change when higher quality evidence becomes available
2A			
Weak recommendation, high-quality evidence	Benefits closely balanced with risks and burden	RCTs without important limitations or overwhelming evidence from observational studies	Weak recommendation, best action may differ depending on the patient, treatment circumstances, or social values
2B			
Weak recommendation, moderate-quality evidence	Benefits closely balanced with risks and burden	RCTs with important limitations (inconsistent results, methodological flaws, indirect, or imprecise) or exceptionally strong evidence from observational studies	Weak recommendation, best action may differ depending on the patient, treatment circumstances, or social values
2C			
Weak recommendation, low-quality or very low-quality evidence	Uncertainty in the estimates of benefits, risks, and burden; benefits, risk, and burden may be closely balanced	Observational studies or case series	Very weak recommendation; alternative treatments may be equally reasonable and merit consideration

**Table 2:** Summary of statements

	Statements
Indications	
Trauma patients	Persistent hypotension, acidosis (pH <7.2), hypothermia (temperature < 34°C) and coagulopathy are strong predictors of the need for abbreviated laparotomy and open abdomen in trauma patients (Grade 2A)
	Risk factors for abdominal compartment syndrome such as damage control surgery, injuries requiring packing and planned reoperation, extreme visceral or retroperitoneal swelling, obesity, elevated bladder pressure when abdominal closure is attempted, abdominal wall tissue loss and aggressive resuscitation are predictors of the necessity for open abdomen in trauma patients (Grade 2B)
	Decompressive laparotomy is indicated in abdominal compartment syndrome if medical treatment has failed after repeated and reliable IAP measurements (Grade 2B)
	The inability to definitively control the source of contamination or the necessity to evaluate the bowel perfusion may be an indicator to leave the abdomen open in post-traumatic bowel injuries (Grade 2B)
Non-trauma patients	Decompressive laparotomy is indicated in abdominal compartment syndrome if medical treatment has failed after repeated and reliable IAP measurements (Grade 2B)
➢ Peritonitis	The open abdomen is an option for emergency surgery patients with severe peritonitis and severe sepsis/septic shock under the following circumstances: abbreviated laparotomy due to the severe physiological derangement, the need for a deferred intestinal anastomosis, a planned second look for intestinal ischemia, persistent source of peritonitis (failure of source control), or extensive visceral oedema with the concern for development of abdominal compartment syndrome (Grade 2C).
➢ Vascular emergencies	The open abdomen should be considered following management of hemorrhagic vascular catastrophes such as ruptured abdominal aortic aneurysm (Grade 1C)
	The open abdomen should be considered following surgical management of acute mesenteric ischemic insults (Grade 2C).
➢ Pancreatitis	In patients with severe acute pancreatitis unresponsive to step-up conservative management surgical decompression and open abdomen open are effective in treating abdominal compartment syndrome (Grade 2C)
	Leaving the abdomen open after surgical necrosectomy for infected pancreatic necrosis is not recommended except in those situations with high risk factors to develop abdominal compartment syndrome (Grade 1C)
Management	
Trauma and non-trauma patients	The role of Damage Control Resuscitation in OA management is fundamental and may influence outcome (Grade 2A)
ICU management	A multidisciplinary approach is encouraged, especially during the patient’s ICU admission (Grade 2A)
	Intra-abdominal pressure measurement is essential in critically ill patients at risk for IAH/ACS (Grade 1B)
	Physiologic optimization is one of the determinants of early abdominal closure (Grade 2A)
	Inotropes and vasopressors administration should be tailored according to patient condition and performed surgical interventions (Grade 1A)
	Fluid balance should be carefully scrutinized (Grade 2A)
	High attention to body temperature should be given, avoiding hypothermia (Grade 2A)
	In presence of coagulopathy or high risk of bleeding the negative pressure should be down regulated balancing the therapeutic necessity of negative pressure and the hemorrhage risk (Grade 2B).
Technique for temporary abdominal closure	Negative pressure wound therapy with continuous fascial traction should be suggested as the preferred technique for temporary abdominal closure (Grade 2B).
	Temporary abdominal closure without negative pressure (e.g. Bogota bag) can be applied in low resource settings accepting a lower delayed fascial closure rate and higher intestinal fistula rate (Grade 2A).
	No definitive recommendations can be given about temporary abdominal closure with NPWT in combination with fluid instillation even if it seems to improve results in trauma patients (Not grades).
Re-exploration before definitive closure	Open abdomen re-exploration should be conducted no later than 24-48 hours after the index and any subsequent operation, with the duration from the previous operation shortening with increasing degrees of patient non-improvement and hemodynamic instability (Grade 1C).
	The abdomen should be maintained open if requirements for on-going resuscitation and/or the source of contamination persists, if a deferred intestinal anastomosis is needed, if there is the necessity for a planned second look for ischemic intestine and lastly if there are concerns about abdominal compartment syndrome development (Grade 2B).
Nutritional support	Open abdomen patients are in a hyper-metabolic condition; immediate and adequate nutritional support is mandatory (Grade 1C).
	Open abdomen techniques result in a significant nitrogen loss that must be replaced with a balanced nutrition regimen (Grade 1C).
	Early enteral nutrition should be started as soon as possible in presence of viable and functional gastrointestinal tract (Grade 1C).
	Enteral nutrition should be delayed in patients with an intestinal tract in discontinuity (temporarily stapled stumps), or in situations of a high output fistula with no possibility to obtain feeding access distal to the fistula or with signs of intestinal obstruction (Grade 2C)
	Oral feeding is not contraindicated and should be used where possible (Grade 2C).
Patient mobilization	To date, no recommendations can be made about early mobilization of patients with open abdomen (Not graded).
Definitive closure	
Trauma and non-trauma patients	Fascia and/or abdomen should be definitively closed as soon as possible (Grade 1C).
Open abdomen definitive closure	Early fascial and/or abdominal definitive closure should be the strategy for management of the open abdomen once any requirements for on-going resuscitation have ceased, the source control has been definitively reached, no concern regarding intestinal viability persist, no further surgical re-exploration is needed and there are no concerns for abdominal compartment syndrome (Grade 1B).
➢ Non-mesh-mediated techniques	Primary fascia closure is the ideal solution to restore the abdominal closure (2A).
	Component separation is an effective technique; however it should not be used for fascial temporary closure. It should be considered only for definitive closure (Grade 2C).
	Planned ventral hernia (skin graft or skin closure only) remains an option for the complicated open abdomen (i.e. in the presence of entero-atmospheric fistula or in cases with a protracted open abdomen due to underlying diseases) or in those settings where no other alternatives are viable (Grade 2C)
➢ Mesh-mediated techniques	The use of synthetic mesh (polypropylene, polytetrafluoruroethylene (PTFE) and polyester products) as a fascial bridge should not be recommended in definitive closure interventions after open abdomen and should be placed only in patients without other alternatives (Grade 1B).
	Biologic meshes are reliable for definitive abdominal wall reconstruction in the presence of a large wall defect, bacterial contamination, comorbidities and difficult wound healing (Grade 2B).
	Non–cross-linked biologic meshes seem to be preferred in sublay position when the linea alba can be reconstructed. (Grade 2B).
	Cross-linked biologic meshes in fascial-bridge position (no linea alba closure) maybe associated with less ventral hernia recurrence (Grade 2B).
	NPWT can be used in combination with biologic mesh to facilitate granulation and skin closure (Grade 2B).
Complications management	
Trauma and non-trauma patients	Preemptive measures to prevent entero-atmospheric fistula and frozen abdomen are imperative (i.e. early abdominal wall closure, bowel coverage with plastic sheets, omentum or skin, no direct application of synthetic prosthesis over bowel loops, no direct application of NPWT on the viscera and deep burying of intestinal anastomoses under bowel loops) (Grade 1C).
	Entero-atmospheric fistula management should be tailored according to patient conditions, fistula output and position and anatomical features (Grade 1C)
	In the presence of entero-atmospheric fistula the caloric intake and protein demands are increased; the nitrogen balance should be evaluated and corrected and protein supplemented (Grade 1C).
	Nutrition should be reviewed and optimized upon recognition of entero-atmospheric fistula (Grade 1C)
	Entero-atmospheric fistula effluent isolation is essential for proper wound healing. Separating the wound into different compartments to facilitate the collection of fistula output is of paramount importance (Grade 2A).
	In the presence of entero-atmospheric fistula in open abdomen, negative pressure wound therapy makes effluent isolation feasible and wound healing achievable (Grade 2A).
	Definitive management of entero-atmospheric fistula should be delayed to after the patient has recovered and the wound completely healed (Grade 1C).

### Indications

#### Trauma patients

*Persistent hypotension, acidosis (pH <7.2), hypothermia (temperature < 34°C) and coagulopathy are strong predictors of the need for abbreviated laparotomy and open abdomen in trauma patients (Grade 2A)*

*Risk factors for abdominal compartment syndrome such as damage control surgery, injuries requiring packing and planned reoperation, extreme visceral or retroperitoneal swelling, obesity, elevated bladder pressure when abdominal closure is attempted, abdominal wall tissue loss and aggressive resuscitation are predictors of the necessity for open abdomen in trauma patients (Grade 2B)*

*Decompressive laparotomy is indicated in abdominal compartment syndrome if medical treatment has failed after repeated and reliable IAP measurements (Grade 2B)*

*The inability to definitively control the source of contamination or the necessity to evaluate bowel perfusion may be an indicator to leave the abdomen open in post-traumatic bowel injuries (Grade 2B)*

Severely injured patients with hemodynamic instability are at higher risk of ACS for several reasons (i.e., aggressive resuscitation, ischemia-reperfusion injury, visceral or retroperitoneal swelling, recurrent bleeding, and intra-peritoneal packing) [9–12].

In fact, the post-traumatic physiological derangements and the consequent DCM expose patients at risk for increased intra-abdominal pressure. Risk factors associated with ACS requiring an OA after trauma, indicating a higher need for OA, are acidosis with pH ≤ 7.2, lactate levels ≥ 5 mmol/L, base deficit (BD) ≥ − 6 in patients older than 55 years or ≥ − 15 in patients younger than 55 years, core temperature ≤ 34 °C, systolic pressure ≤ 70 mmHg, estimated blood loss ≥ 4 L during the operation and/or transfusion requirement ≥ 10 U of packed red blood cells in the pre- or pre- and intraoperative settings, and severe coagulation derangements (INR/PT > 1.5 times normal, with or without a concomitant PTT > 1.5 times normal) [10, 13–17].

Other recognized risk factors for IAH should be kept into consideration: obesity, pancreatitis, hepatic failure/cirrhosis, positive end-expiratory pressure > 10 cm H_2_0, respiratory failure, acute respiratory distress syndrome [18].

All non-surgical treatment should be implemented to prevent or reduce IAH before proceeding to surgical decompression (i.e., nasogastric and colonic decompression, prokinetic agents, adequate patient positioning and avoidance of constrictive dressings, eventual escharotomy and percutaneous decompression, adequate mechanical ventilation, analgesia, sedation and neuromuscular blockade, balanced fluid resuscitation, eventual diuretic therapy and continuous veno-venous hemofiltration/ultrafiltration, and vasoactive medications).

Moreover, failure to definitively control the source of infection at the index operation or the necessity to check bowel perfusion during DCM or abdominal wall tissue loss represents indications to OA management in traumatic abdominal injuries [3, 11].

#### Non-trauma patients

*Decompressive laparotomy is indicated in abdominal compartment syndrome if medical treatment has failed after repeated and reliable IAP measurements (Grade 2B)*

##### Peritonitis

ᅟ

*The open abdomen is an option for emergency surgery patients with severe peritonitis and severe sepsis/septic shock under the following circumstances: abbreviated laparotomy due to severe physiological derangement, the need for a deferred intestinal anastomosis, a planned second look for intestinal ischemia, persistent source of peritonitis (failure of source control), or extensive visceral oedema with the concern for development of abdominal compartment syndrome (Grade 2C).*

Some patients suffering from severe peritonitis may experience a disease progression to septic shock with no room for definitive surgical procedures [3, 19]. In these cases, surgical operation should be abbreviated even in advanced age [20]. In hypotensive patients requiring high-dose vasopressors or inotropes infusion intestinal continuity restoration may be deferred [21]. In incomplete source control or in the presence of visceral edema and/or decreased abdominal wall compliance primary complete fascia closure should not be attempted because of the high risk of IAH/ACS [22]. In all these situations, the abdomen may be left open. However, there is no definitive data regarding the use of the OA in the face of severe peritonitis and therefore, caution should be exercised when using OA in these circumstances.

##### Vascular emergencies

ᅟ

*The open abdomen should be considered following management of hemorrhagic vascular catastrophes such as ruptured abdominal aortic aneurysm (Grade 1C)*

*The open abdomen should be considered following surgical management of acute mesenteric ischemic insults (Grade 2C).*

Up to 20% of patients experiencing a ruptured AAA repair develop ACS. Mortality is high (30–50%) and is almost doubled in presence of ACS [23, 24]. OA reduces the ACS incidence [25]. No definitive indications to OA exist; the relative indications to OA are massive resuscitation, deranged physiology, fascial tension at closure, use of balloon occlusion of the aorta, and blood loss > 5 L [25–27].

Advanced age is not a contraindication to DCM [20].

ACS can occur even after endovascular repair (EVAR), and the major risk factor appears to be massive resuscitation [23]. Risk of graft infection due to OA management has been demonstrated to be low [28].

The use of OA after perfusion restoration in a patient with acute mesenteric ischemia as in occlusive proximal or distal superior mesenteric artery emboli, watershed necrosis after AAA repairs (open or endovascular), and non-occlusive mesenteric ischemia (e.g., post-arrest or resuscitation from shock/arrest) should be considered in case of deranged physiology and bowel edema and necessity to perform a second look or delayed anastomosis [29–31].

Mesenteric venous thrombosis requiring laparotomy does not routinely mandate OA as often as mesenteric ischemia [32]; however, the risk of IAH/ACS imposes attention to IAP.

##### Pancreatitis

ᅟ

*In patients with severe acute pancreatitis unresponsive to step-up conservative management surgical decompression and open abdomen open are effective in treating abdominal compartment syndrome (Grade 2C)*

*Leaving the abdomen open after surgical necrosectomy for infected pancreatic necrosis is not recommended except in those situations with high risk factors to develop abdominal compartment syndrome (Grade 1C)*

MOF is the factor mainly associated with mortality in acute pancreatitis (AP) especially when infected necrosis [33–37] is present. As in many other conditions, secondary IAH/ACS may aggravate MOF in a vicious circle [38]. IAH/ACS should be prevented and treated as far as it is possible with non-surgical measures. Surgical decompression is the last but effective tool; it should not be delayed in case of ACS [4, 39]. Pancreatic necrosis may become infected after the first week [40]. The presence of organ failure, early bacteremia, and the extent of pancreatic necrosis are factors associated with infection [40]. Surgical necrosectomy should be considered when more conservative management as percutaneous drainage fails [41]. In case of necrosectomy, OA may be considered, but it is not mandatory. It should be considered only if risks for IAH/ACS exist.

### Management

#### Trauma and non-trauma patients

##### ICU management

ᅟ

*The role of Damage Control Resuscitation in OA management is fundamental and may influence outcome (Grade 2A)*

*A multidisciplinary approach is encouraged, especially during the patient’s ICU admission (Grade 2A)*

*Intra-abdominal pressure measurement is essential in critically ill patients at risk for IAH/ACS (Grade 1B)*

*Physiologic optimization is one of the determinants of early abdominal closure (Grade 2A)*

*Inotropes and vasopressors administration should be tailored to patient’s condition and performed surgical interventions (Grade 1A)*

*Fluid balance should be carefully scrutinized (Grade 2A)*

*High attention to body temperature should be given, avoiding hypothermia (Grade 2A)*

*In presence of coagulopathy or high risk of bleeding the negative pressure should be down regulated balancing the therapeutic necessity of negative pressure and the hemorrhage risk (Grade 2B).*

The initial management is fundamental. DCR is part of DCM utilized in treating severely injured and severely physiologically deranged patients. It passes through some cornerstone actions as volume resuscitation, reversal of coagulopathy, correction of acidosis, and all the other pertinent resuscitative measures aiming to restore the normal physiology. The fluid status, nutrition, and respiratory mechanics should also be kept into consideration in managing OA. In fact the possibility of recurrent ACS with its related high mortality is to be posed into consideration [42–44].

Abdominal pressure should be measured in all patients at risk of developing IAH/ACS; in fact, it has been demonstrated that clinical examination is inaccurate in diagnosing IAH/ACS [45]. As a general principle, it should be measured every 12 h and every 4–6 h once ACS/IAH has been detected or if organ failure happens.

Physiology optimization is necessary to allow early abdominal closure. In fact, prolonged OA may delay extubation, increase the risk for EAF and frozen abdomen, and increase complications [46].

Multidisciplinary collaboration with all teams managing the patient is required for optimal care of OA patients.

The real extent of heat loss in OA and a temporary abdominal dressing cannot be quantified. It is well known that patient physiology is impaired by hypothermia and its related hypo-perfusion effects such as heart function depression, reduced oxygen delivery, coagulation cascade alteration, and acidosis.

In trauma patients, the “lethal triad” should be rapidly interrupted [47–53].

It is well known that mortality increases in trauma patients with significant core-body temperature drop [54].

Commercial NPWT systems significantly reduce heat loss but the non-commercial ones still maintain a reduced heat isolation capacity. For this reason, the heat loss control is of paramount importance especially in those settings where non-commercial systems are utilized.

During ICU stay, it is important to ensure analgesia over hypnosis and consider multimodal analgesia to reduce opioid infusion, trying to keep the patient “awake” but well adapted to mechanical ventilation. Moreover, protective mechanical ventilation strategies should be adopted.

Fluid balance is important as well in OA management and should be carefully scrutinized to avoid over- or under- resuscitation. Careful monitoring and maintenance of adequate urinary output could help in evaluating adequacy of resuscitation effects. Continuous monitoring of cardiac output (CO), targeting at low/normal values, is essential to avoid fluid overload and vasopressor abuse. If increasing vasopressors induce low CO, and fluid responsiveness is transient, consider to target treatments (included inotropes) to the best compromise between MAP, CO, and fluid amount. High-rate maintenance fluid infusions should be avoided. As a counterpart, whenever possible, frequent, small-volume fluid boluses should be preferred. Hypertonic crystalloid and colloid-based resuscitation seem to decrease the risk of iatrogenic, induce resuscitation, and increase IAP [55]. Daily patient weights may help in evaluating fluid retention.

Inotrope infusion should be balanced keeping in mind the patients’ condition, the performed surgical procedures, and the necessity to prevent further complications due to their overuse [56, 57].

Volumetric-based monitoring technologies can be very useful in hemodynamic evaluation during DCR phases in critically ill patients. In fact, the elevated intra-abdominal and intra-thoracic pressure can impair the real value of the measurements obtained with traditional pressure-based parameters such as pulmonary artery occlusion pressure and central venous pressure [58–60]. The alteration of these parameters can potentially lead to wrong decisions as regards the correct fluid status and as a consequence the necessary amount of fluid to be administered. This balance is essential also to optimize the surgical success of primary fascial closure [12, 61, 62].

##### Technique for temporary abdominal closure

ᅟ

*Negative pressure wound therapy with continuous fascial traction should be suggested as the preferred technique for temporary abdominal closure (Grade 2B).*

*Temporary abdominal closure without negative pressure (e.g. Bogota bag) can be applied in low resource settings accepting a lower delayed fascial closure rate and higher intestinal fistula rate (Grade 2A).*

*No definitive recommendations can be given about temporary abdominal closure with NPWT in combination with fluid instillation even if it seems to improve results in trauma patients (Not graded).*

Several strategies to maintain the OA have been described. They result in different delayed fascial closure rate and EAF risk. In general, negative pressure associated to a dynamic component (mesh-mediated fascial traction or dynamic sutures) allows to reach the best results in terms of delayed fascial closure, but dynamic sutures result more often in fistula [3]. Negative pressure without a dynamic component (Barker’s VAC or commercial products) results in a moderate delayed fascial closure rate and a fistula rate similar to mesh closure without negative pressure [3].

Recent data from the International Register of Open Abdomen (IROA study) showed that different techniques of OA resulted in different results according to the treated disease [63] (trauma and severe peritonitis) and if treated with or without negative pressure in terms of abdominal closure and mortality rate. The results favored the non-negative pressure systems in trauma and negative pressure temporary closure in severe peritonitis patients [46]. Also, recent contradictory data from a single-center RCT showed that NPWT and fluid instillation seemed to improve outcomes in trauma patients in terms of early and primary closure [64].

Another important issue in OA management is the necessity to balance the antimicrobial therapy in relation to positive cultures of intra-abdominal fluids. Two options are generally followed without any strong literature evidence: treating all the cultured organisms (with high proportions of staphylococci, candida, and MDR Gram-negative bacilli including *Pseudomonas*) or a “wait and see” strategy. WSES suggests to follow guidelines for intra-abdominal infections [65].

##### Re-exploration before definitive closure

ᅟ

*Open abdomen re-exploration should be conducted no later than 24-48 hours after the index and any subsequent operation, with the duration from the previous operation shortening with increasing degrees of patient non-improvement and hemodynamic instability (Grade 1C).*

*The abdomen should be maintained open if requirements for on-going resuscitation and/or the source of contamination persists, if a deferred intestinal anastomosis is needed, if there is the necessity for a planned second look for ischemic intestine and lastly if there are concerns about abdominal compartment syndrome development (Grade 2B).*

Indications to re-explore an OA may vary between trauma and non-trauma patients. In general, the patient’s non-improvement possibly is due to an intra-abdominal reason. No definitive data regarding the timing of re-operation in OA patients exist [6, 66]. It is generally recommended that OA patients should be re-explored 24–72 h after the initial or any subsequent surgical intervention [2, 67, 68]. Some data regarding trauma patients showed that the time of re-exploration reduces the primary fascial closure rate of 1.1% for each hour after the first 24 h after the index operation [69]. Moreover, increased complication rate was observed in patients having the first re-operation after 48 h [3, 69].

In non-trauma patients, the indication to re-explore the abdominal cavity are less definite and usually are due to the necessity to continue DCM, to the impossibility to definitively control the source of infection or to the necessity to re-asses the bowel vascularization or lastly, to concerns regarding the possibility of ACS [2, 3, 20, 70].

Even though there is some evidence that OA may be justified in severely injured or physiologically deranged patients with the aim to manipulate the systemic immune response and ameliorate the bio mediator burden, no definitive statement can be made [3, 71–75].

##### Nutritional support

ᅟ

*Open abdomen patients are in a hyper-metabolic condition; immediate and adequate nutritional support is mandatory (Grade 1C).*

*Open abdomen techniques result in a significant nitrogen loss that must be replaced with a balanced nutrition regimen (Grade 1C).*

*Early enteral nutrition should be started as soon as possible in the presence of viable and functional gastrointestinal tract (Grade 1C).*

*Enteral nutrition should be delayed in patients with an intestinal tract in discontinuity (temporarily closed loops ), or in situations of a high output fistula with no possibility to obtain feeding access distal to the fistula or with signs of intestinal obstruction (Grade 2C)*

*Oral feeding is not contraindicated and should be used where possible (Grade 2C).*

Malnutrition is a risk factor for poor outcomes [76]. Critically ill patients with OA are in a hyper-catabolic state with an estimated nitrogen loss of almost 2 g/L of abdominal fluid output. Abdominal fluid evacuation is to be measured in order to adjust nutritional integrations [77]. In case of EAF, nitrogen loss greatly increases. Parenteral nutrition should be started as soon as possible. Once the resuscitation is almost complete and the GI tract is viable, enteral nutrition (EN) should be started. Relative contraindication to EN is a viable bowel shorter than 75 cm [78].

Polymeric formula supplying a daily intake of 20- to 30-kcal/kg non-protein calories with 1.5- to 2.5-g/kg proteins is usually sufficient to maintain a positive nitrogen balance.

EN starting within the first 24–48 h improves wound healing and fascial closure rate, decreases catabolism, reduces pneumonia and fistula rate, preserves GI tract integrity, and finally reduces complications, length of hospital stay, and costs [79–81]. Compared to prolonged total parenteral nutrition, early EN decreases septic complications especially in abdominal trauma and traumatic brain injuries [3, 79, 82, 83].

##### Patient mobilization

ᅟ

*No recommendations can be made about early mobilization of patients with open abdomen (Not graded).*

No definite evidence exists regarding the optimal timing for mobilization of patients with OA [84]. Prolonged bed rest is associated with a significant increase in morbidity. Mobilization occurring within the first 2-5 days of ICU admission is defined “early” [85] and it is associated with positive effects on outcomes [86–90].

OA patients with NPWT may be “early” mobilized by active or passive transfer thanks to the provisional abdominal wall function supplied by NPWT systems [3].

### Definitive closure

#### Open abdomen definitive closure

*Fascia and/or abdomen should be definitively closed as soon as possible (Grade 1C).*

*Early fascial and/or abdominal definitive closure should be the strategy for management of the open abdomen once any requirements for on-going resuscitation have ceased, the source control has been definitively reached, no concern regarding intestinal viability persist, no further surgical re-exploration is needed and there are no concerns for abdominal compartment syndrome (Grade 1B).*

The priority in order to reduce mortality, complications, and length of stay linked to the OA should be the early definitive abdominal closure [10, 91, 92]. Major factors influencing early definitive closure are postoperative ICU management and the TAC technique [93]. Early fascial closure is commonly defined as occurring within 4–7 days from the index operation [21]. In contrast to trauma patients, those affected by abdominal sepsis usually experience a lower rate of early fascial closure [94] even though continuous fascial traction seems to increase this rate [95]. Fascial closure should be attempted as soon as the source of infection is controlled [96].

#### Solutions to definitively close an open abdomen

In case of prolonged OA, fascia retraction and large abdominal wall defects requiring complex abdominal wall reconstruction may occur. In contaminated fields, the complication risk in abdominal wall definitive closure is increased [92, 97–99].

Techniques used to definitively close the abdomen are principally divided into non-mesh and mesh mediated.

##### Non-mesh-mediated closure techniques

ᅟ

*Primary fascia closure is the ideal solution to restore the abdominal closure (2A).*

*Component separation is an effective technique; however it should not be used for fascial temporary closure. It should be considered only for definitive closure (Grade 2C).*

*Planned ventral hernia (skin graft or skin closure only) remains an option for the complicated open abdomen (i.e. in the presence of entero-atmospheric fistula or in cases with a protracted open abdomen due to underlying diseases) or in those settings where no other alternatives are viable (Grade 2C)*

Abdominal component separation should be considered an elective procedure for ventral hernia repair [100]. In fact, it should not be used during the OA management but reserved to the definitive closure interventions. At a delayed time point, very good results reaching up to 75% of fascial closure rate have been reported [101]. The separation of components can be approached anteriorly or posteriorly [102, 103].

Planned ventral hernia represents a valid alternative to cover abdominal viscera and to prevent EAF. In fact, in cases of persistent contamination, several comorbidities or in severely ill patients, with or without sufficient skin to cover the abdominal wall defect, delaying the eventual synthetic prosthetic reconstruction may be a safer option. The decision either to close the skin or to perform vascularized flaps, pedicled flaps in small-/mid-sized defects, or free flaps such as tensor fasciae latae for extensive thoraco-abdominal defects is usually taken, considering the wound conditions, the dimension of the skin defect, and the center facilities [13].

##### Mesh-mediated closure techniques

ᅟ

*The use of synthetic mesh (polypropylene, polytetrafluoruroethylene (PTFE) and polyester products) as a fascial bridge should not be recommended in definitive closure interventions after open abdomen and should be placed only in patients without other alternatives (Grade 1B).*

*Biologic meshes are reliable for definitive abdominal wall reconstruction in the presence of a large wall defect, bacterial contamination, comorbidities and difficult wound healing (Grade 2B).*

*Non–cross-linked biologic meshes seem to be preferred in sublay position when the linea alba can be reconstructed. (Grade 2B).*

*Cross-linked biologic meshes in fascial-bridge position (no linea alba closure) maybe associated with less ventral hernia recurrence (Grade 2B).*

*NPWT can be used in combination with biologic mesh to facilitate granulation and skin closure (Grade 2B).*

Several data exist regarding the abdominal wall closure after OA [104, 105]. Non-absorbable synthetic materials (i.e., polypropylene mesh) in a bridging position (i.e., no linea alba closure), where no native tissue protect viscera, may induce several local side effects (adhesions, erosions, and fistula formation) [106–111]. Synthetic meshes in contaminated fields are not recommended by guidelines in emergency abdominal wall reconstruction [112].

Biological prostheses (BP) were designed to perform as permanent surgical prosthesis in abdominal wall repair, minimizing mesh-related complications. Non-cross-linked biologic mesh is easily integrated, with reduced fibrotic reaction and lesser infection and removal rate [113].

BP can be used as a bridge for large abdominal wall defects [114–127]; however, the long-term outcome of a bridging non-cross-linked BP is laxity of the abdominal wall and a high rate of recurrent ventral hernia [113]. As a consequence, non-cross-linked BP should be used in a sublay position (i.e., with linea alba closure) and cross-linked ones should be preferred when the fascial bridge is needed [128–130]. BP could also tolerate adjunctive NPWT to facilitate wound healing, granulation, and skin closure [131–133].

### Complication management

*Preemptive measures to prevent entero-atmospheric fistula and frozen abdomen are imperative (i.e. early abdominal wall closure, bowel coverage with plastic sheets, omentum or skin, no direct application of synthetic prosthesis over bowel loops, no direct application of NPWT on the viscera and deep burying of intestinal anastomoses under bowel loops) (Grade 1C).*

*Entero-atmospheric fistula management should be tailored according to patient condition, fistula output and position and anatomical features (Grade 1C).*

*In the presence of entero-atmospheric fistula the caloric intake and protein demands are increased; the nitrogen balance should be evaluated and corrected and protein supplemented (Grade 1C).*

*Nutrition should be reviewed and optimized upon recognition of entero-atmospheric fistula (Grade 1C).*

*Entero-atmospheric fistula effluent isolation is essential for proper wound healing. Separating the wound into different compartments to facilitate the collection of fistula output is of paramount importance (Grade 2A).*

*In the presence of entero-atmospheric fistula in open abdomen, negative pressure wound therapy makes effluent isolation feasible and wound healing achievable (Grade 2A).*

*Definitive management of entero-atmospheric fistula should be delayed to after the patient has recovered and the wound completely healed (Grade 1C).*

**Fig. 2:**
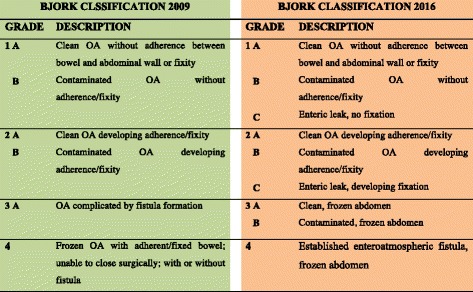
Open Abdomen classification according to Björck et al. [147]

Risk factors for frozen abdomen and EAF in OA are delayed abdominal closure, non-protection of bowel loops during OA, presence of bowel injury and repairs or anastomosis, colon resection during DCS, the large fluid resuscitation volume (> 5 L/24 h), the presence of intra-abdominal sepsis/abscess, and the use of polypropylene mesh directly over the bowel [66, 134–139]. All risk factors often linked as a “vicious cycle” may contribute to the development of frozen abdomen and EAF. Complications increase mortality, length of stays, and costs [140]. Some preemptive measures to prevent this complication are early abdominal wall closure, bowel coverage with plastic sheets, omentum or skin, no direct application of synthetic prosthesis on bowel, no direct application of NPWT on the viscera, and intestinal anastomosis deep buring under bowel loops [73, 141, 142]. EAF can be classified based on the output: low (< 200 mL/day), moderate (200–500 mL/day), and high (> 500 mL/day) [143]; usually, the greater the output, the higher the difficulty in managing the EAF [144, 145]. In EAF management, the definition of characteristics and anatomical features are extremely important in planning the best treatment [146]. The intra-abdominal situation can be classified according to the WSACS classification (Fig. 2) [147]. Nutrition plays a pivotal role in EAF management. While early EN improves outcomes [81, 148–151], it may increase EAF output even if it seems not to impair final outcomes [152, 153]. Spontaneous closure of an EAF is quite impossible; for this reason, the treatment should try to isolate the fistula effluent to allow granulation tissue formation around [3]. Many different effective techniques have been described with no definitive results [138, 144, 145, 154–157]. NPWT in all its variants is effective and the most accepted technique [3]. It often allows EAF isolation, adequate wound management, re-epithelization, and eventual subsequent skin graft with the final conversion of the EAF into a sort of enterostomy. EAF definitive treatment (i.e., fistula closure and abdominal wall reconstruction) should be postponed at least of 6 months and only after the patient and the wound healed completely [3].

## Conclusions

Open abdomen in trauma and non-trauma patients is dramatically effective in facing the deranged physiology of severe injuries or critical illness when no other perceived options exist. Its use remains very controversial and is a matter of great debate, as it is a non-anatomic situation with potential severe side effects and increased resource utilization. Moreover, the lack of definitive data demands carefully tailoring its use to each single patient, taking care to not overuse it. Abdominal closure attempt should be done as soon as the patient can physiologically tolerate it. All possible precautions should be implemented to minimize complications. Results improve proportionate to the clinicians’ team’s experience with the intricacies of open abdomen management.

## Abbreviations

AAST: American Association for the Surgery of Trauma
ACS: Abdominal compartment syndrome
AP: Acute pancreatitis
CO: Cardiac output
DCM: Damage control management
DCR: Damage control resuscitation
DCS: Damage control surgery
EAF: Entero-atmospheric fistula
EN: Enteral nutrition
EVAR: Endovascular repair
GRADE: Grading of Recommendations Assessment, Development and Evaluation
IAH: Intra-abdominal hypertension
IAP: Intra-abdominal pressure
INR: International normalized ratio
MAP: Mean arterial pressure
MOF: Multiple organ failure
NPWT: Negative pressure wound therapy
OA: Open abdomen procedure
PTFE: Polytetrafluoruroethylene
rAAA: Ruptured abdominal aortic aneurysm
RCT: Randomized controlled trial
TAC: Temporal abdominal closure
TEG: Thromboelastography
TPN: Parenteral nutrition
WSACS: World Society Abdominal Compartment Syndrome
WSES: World Society of Emergency Surgery
